# Supplementary material for: COVID-19 vaccine hesitancy and its determinants among sub-Saharan African adolescents
Source: PLOS Glob Public Health. 2022 Oct 5;2(10):e0000611. doi: 10.1371/journal.pgph.0000611 (PMC10022111; doi:10.1371/journal.pgph.0000611)
Supplement: S1 Table — (DOCX) [file pgph.0000611.s001.docx]

**S1 Table** COVID-19 vaccine hesitancy by age among adolescents in a phone-based survey in five sub-Saharan African countries, 2021^1^

|  | Burkina Faso | | Ethiopia | | Ghana | Nigeria | | Tanzania | |
| --- | --- | --- | --- | --- | --- | --- | --- | --- | --- |
|  | Rural | Urban | Rural | Urban | Rural | Rural | Urban | Rural | Urban |
|  | Nouna | Ouagadougou | Kersa | Addis Ababa | Kintampo | Ibadan | Lagos | Dodoma | Dar es Salaam |
| Number of adolescents, *N* | 309 | 281 | 274 | 268 | 300 | 278 | 332 | 318 | 302 |
| All ages | 30.7 | 31.6 | 13.7 | 36.9 | 47.9 | 22.5 | 64.7 | 88.0 | 75.6 |
| Age: 10-14 years (young adolescents) | 23.1 | 27.2 | 15.2 | 24.0 | 46.3 | 15.2 | 58.6 | 87.3 | 80.2 |
| 10 years | 0 | NA | NA | 0 | 46.2 | 0 | 100 | 91.7 | 100 |
| 11 years | 0 | 30.0 | 11.1 | 20.0 | 72.7 | NA | 55.6 | 95.7 | 80.8 |
| 12 years | 23.1 | 9.5 | 25.0 | 37.5 | 45.5 | 50.0 | 66.7 | 90.9 | 80.7 |
| 13 years | 30.8 | 36.0 | 22.2 | 23.5 | 46.7 | 18.2 | 60.0 | 79.6 | 79.1 |
| 14 years | 18.2 | 32.0 | 0 | 21.1 | 33.3 | 10.5 | 54.2 | 87.9 | 69.1 |
| Age: 15-19 years (older adolescents) | 33.8 | 33.7 | 13.3 | 39.9 | 48.6 | 23.7 | 66.0 | 88.7 | 69.5 |
| 15 years | 26.7 | 37.8 | 15.0 | 28.1 | 40.6 | 24.4 | 71.4 | 87.5 | 86.8 |
| 16 years | 26.3 | 41.0 | 0 | 30.8 | 39.1 | 25.9 | 71.1 | 85.7 | 61.5 |
| 17 years | 36.7 | 34.5 | 25.0 | 44.2 | 55.2 | 15.8 | 60.9 | 94.7 | 65.0 |
| 18 years | 26.1 | 17.9 | 14.3 | 42.0 | 59.2 | 21.6 | 52.9 | 94.4 | 52.4 |
| 19 years | 41.4 | 33.3 | 6.9 | 45.2 | 43.5 | 29.7 | 64.3 | 85.7 | 69.6 |

^1^ Values are percentages out of each age or age range. COVID-19 vaccine hesitancy was defined as a response of definitely not getting the COVID-19 vaccine, or a response of maybe, unsure, or undecided on whether to get the COVID-19 vaccine if it were available now. NA indicates lack of adolescents for a specific age in the sample.
