# Supplementary material for: COVID-19 vaccine hesitancy and its determinants among sub-Saharan African adolescents
Source: PLOS Glob Public Health. 2022 Oct 5;2(10):e0000611. doi: 10.1371/journal.pgph.0000611 (PMC10022111; doi:10.1371/journal.pgph.0000611)
Supplement: S2 Table — (DOCX) [file pgph.0000611.s002.docx]

**S2 Table** Perceptions of the COVID-19 vaccine among adolescents in a phone-based survey in five sub-Saharan African countries, 2021^1^

|  | Burkina Faso | | Ethiopia | | Ghana | Nigeria | | Tanzania | | Total |
| --- | --- | --- | --- | --- | --- | --- | --- | --- | --- | --- |
|  | Rural | Urban | Rural | Urban | Rural | Rural | Urban | Rural | Urban |  |
|  | Nouna | Ouagadougou | Kersa | Addis Ababa | Kintampo | Ibadan | Lagos | Dodoma | Dar es Salaam |  |
| Number of adolescents, *N* | 309 | 281 | 274 | 268 | 300 | 278 | 332 | 318 | 302 | 2662 |
| Perceived safety of the COVID-19 vaccine in general,^2,3^ *N* (%) |  |  |  |  |  |  |  |  |  |  |
| Very safe | 135 (43.7) | 111 (39.8) | 205 (74.8) | 45 (16.8) | 67 (22.3) | 155 (55.8) | 88 (26.5) | 46 (14.5) | 92 (30.5) | 944 (35.5) |
| Somewhat safe | 68 (22.0) | 54 (19.4) | 3 (1.1) | 162 (60.5) | 69 (23.0) | 47 (16.9) | 88 (26.5) | 76 (23.9) | 57 (18.9) | 624 (23.5) |
| Not very safe | 50 (16.2) | 51 (18.3) | 5 (1.8) | 21 (7.8) | 42 (14.0) | 22 (7.9) | 82 (24.7) | 80 (25.2) | 28 (9.3) | 381 (14.3) |
| Not safe at all | 5 (1.6) | 15 (5.4) | 2 (0.7) | 22 (8.2) | 16 (5.3) | 10 (3.6) | 28 (8.4) | 19 (6.0) | 54 (17.9) | 171 (6.4) |
| Do not know | 51 (16.5) | 48 (17.2) | 59 (21.5) | 18 (6.7) | 106 (35.3) | 44 (15.8) | 46 (13.9) | 97 (30.5) | 71 (23.5) | 540 (20.3) |
| Perceived safety of the COVID-19 vaccine among children and adolescents,^2,4^ *N* (%) |  |  |  |  |  |  |  |  |  |  |
| Very safe | 131 (42.4) | 84 (31.0) | 210 (76.6) | 61 (22.8) | 50 (16.7) | 137 (49.3) | 86 (25.9) | 39 (12.3) | 84 (27.8) | 882 (33.3) |
| Somewhat safe | 65 (21.0) | 61 (22.5) | 2 (0.7) | 122 (45.5) | 59 (19.7) | 56 (20.1) | 82 (24.7) | 66 (20.8) | 47 (15.6) | 560 (21.1) |
| Not very safe | 53 (17.2) | 55 (20.3) | 6 (2.2) | 37 (13.8) | 48 (16.0) | 27 (9.7) | 83 (25.0) | 84 (26.5) | 28 (9.3) | 421 (15.9) |
| Not safe at all | 7 (2.3) | 19 (7.0) | 2 (0.7) | 23 (8.6) | 27 (9.0) | 11 (4.0) | 29 (8.7) | 31 (9.8) | 66 (21.9) | 215 (8.1) |
| Do not know | 53 (17.2) | 52 (19.2) | 54 (19.7) | 25 (9.3) | 116 (38.7) | 47 (16.9) | 52 (15.7) | 97 (30.6) | 77 (25.5) | 573 (21.6) |
| Perceived effectiveness of the COVID-19 vaccine in general,^2,5^ *N* (%) |  |  |  |  |  |  |  |  |  |  |
| Very effective | 136 (44.0) | 112 (40.7) | 204 (74.5) | 60 (22.4) | 63 (21.0) | 147 (52.9) | 84 (25.3) | 48 (15.2) | 71 (23.5) | 925 (34.9) |
| Somewhat effective | 65 (21.0) | 54 (19.6) | 16 (5.8) | 134 (50.0) | 58 (19.3) | 61 (21.9) | 77 (23.2) | 75 (23.8) | 57 (18.9) | 597 (22.5) |
| Not very effective | 42 (13.6) | 35 (12.7) | 4 (1.5) | 23 (8.6) | 31 (10.3) | 22 (7.9) | 70 (21.1) | 80 (25.4) | 31 (10.3) | 338 (12.7) |
| Not effective at all | 6 (1.9) | 15 (5.5) | 2 (0.7) | 16 (6.0) | 16 (5.3) | 5 (1.8) | 25 (7.5) | 19 (6.0) | 54 (17.9) | 158 (6.0) |
| Do not know | 60 (19.4) | 59 (21.5) | 48 (17.5) | 35 (13.1) | 132 (44.0) | 43 (15.5) | 76 (22.9) | 93 (29.5) | 89 (29.5) | 635 (23.9) |
| Perceived side effects of the COVID-19 vaccine,^6,7^ *N* (%) |  |  |  |  |  |  |  |  |  |  |
| No side effects | 52 (16.8) | 70 (24.9) | 121 (44.2) | 98 (36.6) | 49 (16.3) | 101 (36.3) | 50 (15.1) | 99 (31.3) | 88 (29.1) | 728 (27.4) |
| Fever | 64 (20.7) | 39 (13.9) | 1 (0.4) | 56 (20.9) | 33 (11.0) | 45 (16.2) | 79 (23.8) | 3 (1.0) | 25 (8.3) | 345 (13.0) |
| Body ache including sore arm | 59 (19.1) | 18 (6.4) | 7 (2.6) | 96 (35.8) | 38 (12.7) | 56 (20.1) | 88 (26.5) | 3 (1.0) | 13 (4.3) | 378 (14.2) |
| Nausea | 54 (17.5) | 20 (7.1) | 1 (0.4) | 38 (14.2) | 10 (3.3) | 6 (2.2) | 19 (5.7) | 0 (0.0) | 6 (2.0) | 154 (5.8) |
| Tiredness/exhaustion | 62 (20.1) | 36 (12.8) | 7 (2.6) | 67 (25.0) | 24 (8.0) | 55 (19.8) | 64 (19.3) | 0 (0.0) | 10 (3.3) | 325 (12.2) |
| Do not know any | 183 (59.2) | 150 (53.4) | 141 (51.5) | 34 (12.7) | 179 (59.7) | 99 (35.6) | 121 (36.5) | 203 (64.2) | 119 (39.4) | 1229 (46.2) |

^1^ Values are counts (percentages) for categorical variables.

^2^ Percentages may not add up to 100% due to rounding.

^3^ Missing for 2 adolescents in Ouagadougou.

^4^ Missing for 10 adolescents in Ouagadougou and 1 adolescent in Dodoma.

^5^ Missing for 6 adolescents in Ouagadougou and 3 adolescents in Dodoma.

^6^ Counts and percentages do not add up to the total because the selection of multiple reasons was allowed.

^7^ Missing for 2 adolescents in Dodoma.
