# Supplementary material for: COVID-19 vaccine hesitancy and its determinants among sub-Saharan African adolescents
Source: PLOS Glob Public Health. 2022 Oct 5;2(10):e0000611. doi: 10.1371/journal.pgph.0000611 (PMC10022111; doi:10.1371/journal.pgph.0000611)
Supplement: S3 Table — (DOCX) [file pgph.0000611.s003.docx]

**S3 Table** Determinants of the willingness to receive the COVID-19 vaccine among adolescents in a phone-based survey in five sub-Saharan African countries, 2021^1^

|  | Burkina Faso | | Ethiopia | | Ghana | Nigeria | | Tanzania | | Total |
| --- | --- | --- | --- | --- | --- | --- | --- | --- | --- | --- |
|  | Rural | Urban | Rural | Urban | Rural | Rural | Urban | Rural | Urban |  |
|  | Nouna | Ouagadougou | Kersa | Addis Ababa | Kintampo | Ibadan | Lagos | Dodoma | Dar es Salaam |  |
| Number of adolescents, *N* | 309 | 281 | 274 | 268 | 300 | 278 | 332 | 318 | 302 | 2662 |
| Willingness affected by the vaccine’s country of origin,^2,3^ *N* (%) |  |  |  |  |  |  |  |  |  |  |
| No | 224 (72.5) | 157 (55.9) | 176 (64.2) | 189 (70.5) | 183 (61.4) | 225 (80.9) | 168 (50.8) | 210 (68.2) | 209 (69.2) | 1741 (65.7) |
| Yes | 75 (24.3) | 113 (40.2) | 53 (19.3) | 74 (27.6) | 88 (29.5) | 41 (14.8) | 149 (45.0) | 49 (15.9) | 66 (21.9) | 708 (26.7) |
| Do not know | 10 (3.2) | 11 (3.9) | 45 (16.4) | 5 (1.9) | 27 (9.1) | 12 (4.3) | 14 (4.2) | 49 (15.9) | 27 (8.9) | 200 (7.6) |
| Willing to receive COVID-19 vaccine developed by the country,^4,5^ *N* (%) |  |  |  |  |  |  |  |  |  |  |
| United States | 36 (42.4) | 38 (30.7) | 17 (17.4) | 27 (34.2) | 53 (46.9) | 30 (56.6) | 109 (66.9) | 1 (1.1) | 14 (15.1) | 325 (36.2) |
| China | 33 (38.8) | 46 (37.1) | 25 (25.5) | 24 (30.4) | 22 (19.5) | 5 (9.4) | 28 (17.2) | 1 (1.1) | 9 (9.7) | 193 (21.5) |
| Russia | 26 (30.6) | 27 (21.8) | 5 (5.1) | 11 (13.9) | 39 (34.5) | 3 (5.7) | 10 (6.1) | 0 (0.0) | 1 (1.1) | 122 (13.6) |
| India | 15 (17.7) | 24 (19.4) | 3 (3.1) | 3 (3.8) | 29 (25.7) | 3 (5.7) | 18 (11.0) | 1 (1.1) | 2 (2.2) | 98 (10.9) |
| Europe | 18 (21.2) | 17 (13.7) | 8 (8.2) | 11 (13.9) | 29 (25.7) | 12 (22.6) | 33 (20.3) | 0 (0.0) | 3 (3.2) | 131 (14.6) |
| Do not know | 24 (28.2) | 11 (8.9) | 45 (45.9) | 17 (21.5) | 28 (24.8) | 11 (20.8) | 27 (16.6) | 56 (61.5) | 34 (36.6) | 253 (28.1) |
| Willingness of receiving a COVID-19 vaccine developed or tested in Africa,^2,6^ *N* (%) |  |  |  |  |  |  |  |  |  |  |
| Would not affect willingness | 167 (54.2) | 109 (38.8) | 166 (60.6) | 170 (63.4) | 122 (40.7) | 200 (71.9) | 178 (53.6) | 196 (65.3) | 179 (59.3) | 1487 (56.3) |
| Would decrease willingness | 13 (4.2) | 23 (8.2) | 3 (1.1) | 38 (14.2) | 15 (5.0) | 24 (8.6) | 57 (17.2) | 6 (2.0) | 0 (0.0) | 179 (6.8) |
| Would increase willingness | 111 (36.0) | 134 (47.7) | 66 (24.1) | 53 (19.8) | 143 (47.7) | 42 (15.1) | 85 (25.6) | 55 (18.3) | 103 (34.1) | 792 (30.0) |
| Do not know | 17 (5.5) | 15 (5.3) | 39 (14.2) | 7 (2.6) | 20 (6.7) | 12 (4.3) | 12 (3.6) | 43 (14.3) | 20 (6.6) | 185 (7.0) |
| Willingness affected by individuals or groups,^4^ *N* (%) |  |  |  |  |  |  |  |  |  |  |
| Parents or family members | 248 (80.3) | 208 (74.0) | 145 (52.9) | 93 (34.7) | 231 (77.0) | 210 (75.5) | 241 (72.6) | 42 (13.2) | 130 (43.1) | 1548 (58.2) |
| Religious leaders | 143 (46.3) | 114 (40.6) | 91 (33.2) | 74 (27.6) | 161 (53.7) | 91 (32.7) | 146 (44.0) | 68 (21.4) | 74 (24.5) | 962 (36.1) |
| Community/tribal leaders | 113 (36.6) | 99 (35.2) | 73 (26.6) | 78 (29.1) | 141 (47.0) | 55 (19.8) | 63 (19.0) | 21 (6.6) | 19 (6.3) | 662 (24.9) |
| Political leaders | 63 (20.4) | 122 (43.4) | 67 (24.5) | 39 (14.6) | 115 (38.3) | 44 (15.8) | 53 (16.0) | 27 (8.5) | 47 (15.6) | 577 (21.7) |
| Celebrities or social media influencers | 38 (12.3) | 66 (23.5) | 20 (7.3) | 51 (19.0) | 83 (27.7) | 45 (16.2) | 61 (18.4) | 12 (3.8) | 30 (9.9) | 406 (15.3) |
| Healthcare workers | 211 (68.3) | 215 (76.5) | 143 (52.2) | 148 (55.2) | 222 (74.0) | 210 (75.5) | 237 (71.4) | 136 (42.8) | 81 (26.8) | 1603 (60.2) |
| Schoolteachers | 168 (54.4) | 161 (57.3) | 140 (51.1) | 75 (28.0) | 164 (54.7) | 149 (53.6) | 98 (29.5) | 110 (34.6) | 164 (54.3) | 1229 (46.2) |

^1^ Values are counts (percentages) for categorical variables.

^2^ Percentages may not add up to 100% due to rounding.

^3^ Missing for 1 adolescent in Lagos, 10 adolescents in Dodoma, and 2 adolescents in Kintampo.

^4^ Counts and percentages do not add up to the total because the selection of multiple reasons was allowed.

^5^ Counts and percentages are among adolescents whose willingness to take a COVID-19 vaccine may be affected by the vaccine’s country of origin. Missing for 7 adolescents in Dodoma and 2 adolescents in Kintampo.

^6^ Missing for 1 adolescent in Nouna and 18 adolescents in Dodoma.
