# Supplementary material for: COVID-19 vaccine hesitancy and its determinants among sub-Saharan African adolescents
Source: PLOS Glob Public Health. 2022 Oct 5;2(10):e0000611. doi: 10.1371/journal.pgph.0000611 (PMC10022111; doi:10.1371/journal.pgph.0000611)
Supplement: S4 Table — (DOCX) [file pgph.0000611.s004.docx]

**S4 Table** Trusted information sources and expectations about the COVID-19 vaccine among adolescents in a phone-based survey in five sub-Saharan African countries, 2021^1^

|  | Burkina Faso | | Ethiopia | | Ghana | Nigeria | | Tanzania | | Total |
| --- | --- | --- | --- | --- | --- | --- | --- | --- | --- | --- |
|  | Rural | Urban | Rural | Urban | Rural | Rural | Urban | Rural | Urban |  |
|  | Nouna | Ouagadougou | Kersa | Addis Ababa | Kintampo | Ibadan | Lagos | Dodoma | Dar es Salaam |  |
| Number of adolescents, *N* | 309 | 281 | 274 | 268 | 300 | 278 | 332 | 318 | 302 | 2662 |
| Trusted information sources regarding the COVID-19 vaccine,^2^ *N* (%) |  |  |  |  |  |  |  |  |  |  |
| Television, radio, or newspaper | 285 (92.2) | 264 (94.3) | 234 (85.4) | 226 (84.6) | 230 (77.2) | 251 (90.3) | 244 (73.5) | 259 (82.5) | 261 (87.3) | 2254 (85.0) |
| Social media | 101 (32.8) | 107 (38.1) | 34 (12.4) | 77 (28.7) | 126 (42.0) | 165 (59.4) | 105 (31.6) | 79 (25.2) | 55 (18.2) | 849 (32.0) |
| Internet | 108 (35.0) | 142 (50.7) | 43 (15.7) | 86 (32.1) | 168 (56.0) | 164 (59.2) | 125 (37.8) | 71 (22.7) | 61 (20.2) | 968 (36.5) |
| Friends/family | 208 (67.5) | 227 (81.1) | 167 (61.0) | 177 (66.0) | 187 (62.3) | 239 (86.0) | 220 (66.3) | 160 (51.1) | 172 (57.0) | 1757 (66.2) |
| Religious bodies/leaders | 196 (63.4) | 192 (68.3) | 180 (65.9) | 173 (64.8) | 193 (64.3) | 138 (49.6) | 191 (57.5) | 130 (41.5) | 156 (51.7) | 1549 (58.3) |
| Medical professionals | 252 (81.8) | 247 (88.2) | 247 (90.2) | 240 (89.6) | 253 (84.3) | 258 (92.8) | 293 (88.3) | 197 (62.3) | 206 (68.2) | 2193 (82.5) |
| Schoolteachers | 204 (66.0) | 213 (75.8) | 247 (90.2) | 159 (59.3) | 210 (70.2) | 209 (75.2) | 153 (46.1) | 153 (49.4) | 206 (68.2) | 1754 (66.1) |
| Government communications/announcements | 224 (72.5) | 251 (90.0) | 229 (83.6) | 229 (85.5) | 236 (78.7) | 242 (87.4) | 230 (69.3) | 68 (21.9) | 233 (77.2) | 1942 (73.3) |
| Willing to participate in a COVID-19 vaccine trial,^3,4^ *N* (%) |  |  |  |  |  |  |  |  |  |  |
| No | 134 (43.5) | 153 (54.5) | 81 (29.6) | 147 (55.1) | 228 (76.0) | 88 (31.7) | 261 (78.6) | 254 (82.2) | 213 (70.5) | 1559 (58.8) |
| Yes | 138 (44.8) | 120 (42.7) | 182 (66.4) | 114 (42.7) | 64 (21.3) | 151 (54.3) | 59 (17.8) | 44 (14.2) | 72 (23.8) | 944 (35.6) |
| Do not know | 36 (11.7) | 8 (2.9) | 11 (4.0) | 6 (2.3) | 8 (2.7) | 39 (14.0) | 12 (3.6) | 11 (3.6) | 17 (5.6) | 148 (5.6) |
| Perceived timeline of availability of a COVID-19 vaccine,^3,5^ *N* (%) |  |  |  |  |  |  |  |  |  |  |
| Never | 30 (9.7) | 72 (25.6) | 0 (0.0) | 59 (22.0) | 21 (7.1) | 15 (5.4) | 33 (9.9) | 215 (70.0) | 125 (41.4) | 570 (21.5) |
| Already received | 5 (1.6) | 2 (0.7) | 2 (0.7) | 4 (1.5) | 3 (1.0) | 37 (13.3) | 2 (0.6) | 0 (0.0) | 3 (1.0) | 58 (2.2) |
| Before the end of 2021 | 99 (32.0) | 60 (21.4) | 69 (25.3) | 71 (26.5) | 58 (19.5) | 80 (28.8) | 70 (21.1) | 15 (4.9) | 39 (12.9) | 561 (21.2) |
| During the first 6 months of 2022 | 14 (4.5) | 23 (8.2) | 30 (11.0) | 38 (14.2) | 5 (1.7) | 17 (6.1) | 33 (9.9) | 7 (2.3) | 9 (3.0) | 176 (6.7) |
| During the last 6 months of 2022 | 7 (2.3) | 7 (2.5) | 5 (1.8) | 26 (9.7) | 9 (3.0) | 2 (0.7) | 9 (2.7) | 0 (0.0) | 3 (1.0) | 68 (2.6) |
| 2023 or later | 8 (2.6) | 28 (10.0) | 0 (0.0) | 29 (10.8) | 19 (6.4) | 8 (2.9) | 13 (3.9) | 2 (0.7) | 17 (5.6) | 124 (4.7) |
| Do not know | 146 (47.3) | 89 (31.7) | 167 (61.2) | 41 (15.3) | 183 (61.4) | 119 (42.8) | 172 (51.8) | 68 (22.2) | 106 (35.1) | 1091 (41.2) |
| Believing that people should continue following COVID-19 preventative guidelines even after vaccines are available,^3,6^ *N* (%) |  |  |  |  |  |  |  |  |  |  |
| No | 61 (19.8) | 12 (4.3) | 17 (6.2) | 22 (8.2) | 22 (7.3) | 3 (1.1) | 31 (9.3) | 13 (4.1) | 19 (6.3) | 200 (7.5) |
| Yes | 241 (78.3) | 265 (94.3) | 235 (85.8) | 246 (91.8) | 270 (90.0) | 262 (95.3) | 291 (87.7) | 298 (94.0) | 281 (93.1) | 2389 (89.9) |
| Do not know | 6 (2.0) | 4 (1.4) | 22 (8.0) | 0 (0.0) | 8 (2.7) | 10 (3.6) | 10 (3.0) | 6 (1.9) | 2 (0.7) | 68 (2.6) |

^1^ Values are counts (percentages) for categorical variables.

^2^ Counts and percentages do not add up to the total because the selection of multiple reasons was allowed.

^3^ Percentages may not add up to 100% due to rounding.

^4^ Missing for 1 adolescent in Nouna, 1 adolescent in Addis Ababa, and 9 adolescents in Dodoma.

^5^ Missing for 1 adolescent in Kersa, 11 adolescents in Dodoma, and 2 adolescents in Kintampo.

^6^ Missing for 1 adolescent in Nouna, 3 adolescents in Ibadan, and 1 adolescent in Dodoma.
