# Supplementary material for: COVID-19 vaccine hesitancy and its determinants among sub-Saharan African adolescents
Source: PLOS Glob Public Health. 2022 Oct 5;2(10):e0000611. doi: 10.1371/journal.pgph.0000611 (PMC10022111; doi:10.1371/journal.pgph.0000611)
Supplement: Attachment — Submitted filename: PGH_Responses_to_Reviewers_080522.docx [file pgph.0000611.s005.docx]

We thank the editor and the two reviewers for their time and effort in reviewing our manuscript. We appreciate the important input and have incorporated the suggested revisions. Please find below our point-by-point responses to the editor’s and reviewers’ comments.

Additional Editor Comments (if provided):

The two reviewers that reviewed the manuscript have made important recommendation based on their expertise and observations of what this manuscript requires to express the underlying thesis it seeks to argue. The study covers an important theme of contemporary public health issue appearing in learned journals which readers may find interesting to read and learn important lessons by the time corrections are effected and manuscript is published. We at PLoS Global Public Health are encouraged and eager to bring this work to the readership of our discerning academic community hence these suggested revisions are essential for this to happen.

1. The title appear good and addresses contemporary issue of wide impact.

2. The population of interest is broad covering a wide area of at-risk population, this is good.

3. The study is premised on behaviour diagnosis of the likelihood of accepting to receive COVID-19 vaccine based on predictive value of the relationship between set of predictors variables.

However there are equally observed weaknesses in not conceptualizing the study appropriately in theory-grounded behavioral science of health-seeking operationalized in the health belief model. The manuscript would be strengthened id this is addressed, since the word "determinants" and the variables in the health belief model includes knowledge, attitude and perception, makes it easy to include a paragraph of theoretical and conceptual clarification of how these variables may provide elucidation of the dynamics associated with the problem phenomenon. Remember, the health belief model is the theory underpinning the identified problem phenomenon.

The study would not contribute much if it has not attempted to elucidation the dynamics involved in the findings from the data guided by the theory.

We thank the editor and the reviewers for pointing out this weakness in our manuscript. We agree that the study would be strengthened if conceptualized in a theoretical underpinning (e.g., the health belief model). To address this shortcoming, we have added the discussion on the theoretical underpinning (lines 106-111 in tracked changes): “Based on the health belief model, the likelihood of receiving COVID-19 vaccines may be affected by various factors, such as perceived threats from COVID-19 infection, evaluation of COVID-19 vaccines, cues to action received from the media and other individuals, and modifying sociodemographic characteristics. Behaviors toward vaccines may also be driven by cultural, social, historical, and political factors, which likely vary across cultural and geographical settings.” We have also created a figure for the conceptual framework based on the health belief model (**Fig 1**). Based on Fig 1 and as added in lines 209-215 in tracked changes: “Data collection and statistical analysis were informed by the theoretical underpinning of the health belief model (**Fig 1**). Based on this conceptual framework, COVID-19 vaccine hesitancy among adolescents may be driven by perceived susceptibility to COVID-19 exposure, perceived severity of COVID-19 infection, perceived effectiveness and safety of COVID-19 vaccines, and cues to action received from media and other influencers (e.g., family members, teachers, healthcare providers). Several individual-level sociodemographic characteristics may also drive willingness to receive COVID-19 vaccines.”

Other issues observed require minor attention and revisions.

Reviewers' comments:

Reviewer's Responses to Questions

**Comments to the Author**

1. Does this manuscript meet PLOS Global Public Health’s [publication criteria](https://journals.plos.org/globalpublichealth/s/criteria-for-publication)? Is the manuscript technically sound, and do the data support the conclusions? The manuscript must describe methodologically and ethically rigorous research with conclusions that are appropriately drawn based on the data presented.

Reviewer #1: Yes

Reviewer #2: Yes

2. Has the statistical analysis been performed appropriately and rigorously?

Reviewer #1: Yes

Reviewer #2: Yes

3. Have the authors made all data underlying the findings in their manuscript fully available (please refer to the Data Availability Statement at the start of the manuscript PDF file)?

The [PLOS Data policy](https://journals.plos.org/globalpublichealth/s/data-availability) requires authors to make all data underlying the findings described in their manuscript fully available without restriction, with rare exception. The data should be provided as part of the manuscript or its supporting information, or deposited to a public repository. For example, in addition to summary statistics, the data points behind means, medians and variance measures should be available. If there are restrictions on publicly sharing data—e.g. participant privacy or use of data from a third party—those must be specified.

Reviewer #1: Yes

Reviewer #2: Yes

4. Is the manuscript presented in an intelligible fashion and written in standard English?

PLOS Global Public Health does not copyedit accepted manuscripts, so the language in submitted articles must be clear, correct, and unambiguous. Any typographical or grammatical errors should be corrected at revision, so please note any specific errors here.

Reviewer #1: Yes

Reviewer #2: Yes

5. Review Comments to the Author

Please use the space provided to explain your answers to the questions above. You may also include additional comments for the author, including concerns about dual publication, research ethics, or publication ethics. (Please upload your review as an attachment if it exceeds 20,000 characters)

Reviewer #1: The title for the study appears appropriate, the study is important in so many ways but most significantly, it describes population preventive health issues of great concern, and it is premised on behaviour diagnosis of the likelihood of accepting to receive COVID-19 vaccine based on predictive value of the relationship between set of predictors including information resources which, to an extent, was left out and the outcome variable in the study.

We thank reviewer 1 for reviewing our manuscript and for the kind words on the importance of this study. The reviewer's comments and suggestions are valuable, and we hope we have adequately addressed the reviewer's concerns. Please find below our point-by-point responses to the reviewer's input.

This reviewer observed that in the theoretical conceptualization, the study has overlooked the value of establishing its thesis on adequate behavioural grounding by mere selection of two variables perception and attitudinal disposition without recourse to sound theoretical basis, for instance, how certain behaviour theories may be well positioned in elucidating the dynamics of the problem phenomenon- COVID-19 vaccine hesitancy among sub-Saharan African adolescents by applying the principles of health-seeking behaviour and it typologies operationalized by the health belief model which clearly identifies health knowledge gained from information adequacy from the environment among others. The absence of proper scientific foundation in conceptualizing this study has weakened its argument. The study lacked adequate conceptual framework to tailor the research questions, what the research is hypothesizing, the development of the instrument for data collection.

We thank the reviewer for pointing out this weakness in our manuscript. We agree that the study would be further strengthened if conceptualized in a health belief model as a theoretical underpinning. To address this weakness, we have added the discussion on the theoretical underpinning (lines 106-111 in tracked changes): “Based on the health belief model, the likelihood of receiving COVID-19 vaccines may be affected by various factors, such as perceived threats from COVID-19 infection, evaluation of COVID-19 vaccines, cues to action received from the media and other individuals, and modifying sociodemographic characteristics. Behaviors toward vaccines may also be driven by cultural, social, historical, and political factors, which likely vary across cultural and geographical settings.”

We have also created a figure for the conceptual framework based on the health belief model (**Fig 1**). Based on Fig 1 and as added in lines 209-215 in tracked changes: “Data collection and statistical analysis were informed by the theoretical underpinning of the health belief model (**Fig 1**). Based on this conceptual framework, COVID-19 vaccine hesitancy among adolescents may be driven by perceived susceptibility to COVID-19 exposure, perceived severity of COVID-19 infection, perceived effectiveness and safety of COVID-19 vaccines, and cues to action received from media and other influencers (e.g., family members, teachers, healthcare providers). Several individual-level sociodemographic characteristics may also drive willingness to receive COVID-19 vaccines.”

A. OBSERVATIONS: This study is such an important study which should hold important answers within its thesis and narrative. Inevitably, there are major issues to be addressed to strengthen the argument of the authors as to what constitute the determinants of COVID-19 vaccine hesitancy among sub-Saharan African adolescents and the levels –(in terms of measures of these variables and constructs and not merely frequency distribution counts) of the variables; more of synthesizing standardized scores to compare across the multi-country study.

i. The Abstract. The countries mentioned in lines 52-53, were study locations and participants selected on the basis of random balloting or by convenience sampling and how was 300 adolescent participants computed as sample size? Clarification on critical appraisal providing the choice of variables for considerations in selecting appropriate behaviour theories related to variable of interest, and the corresponding antecedent variables which would provide possible elucidation of the problem phenomenon.

We thank the reviewer for raising this very pertinent point regarding our sampling strategy. The study countries and study sites were selected based on research team capacity and infrastructure from existing collaborations in the African Research, Implementation Science and Education (ARISE) Network. The target sample size was determined based on budget constraints, and we did not conduct a formal sample size calculation to determine the target sample size. We have now clarified this in lines 147-151 in tracked changes. Because the study areas were selected based on existing connections and infrastructure, and the adolescents were not selected probabilistically, the results from this study may not be generalized to all adolescents in the country, a limitation we acknowledged in lines 491-494. Nevertheless, we attempted to increase the generalizability by calling adolescents randomly from each sampling frame (line 146). The generalizability of the sample was also increased by including countries and areas geographically spread across sub-Saharan Africa (lines 494-496). Therefore, we hope the reviewer will agree that our analysis nonetheless represents an important (albeit imperfect) characterization of COVID-19 vaccine hesitancy among sub-Saharan African adolescents (lines 496-497).

Line 57: Why not state logistic regression analysis instead of Log-binomial models. Is this a different statistical analysis?

We thank the reviewer for raising this point regarding statistical models. Logistic regression models compute odds ratios, whereas log-binomial models compute prevalence ratios (in cross-sectional studies) and risk ratios (in cohort studies). For rare outcomes (commonly used rule of thumb: an incidence of less than 10%), the odds ratios from logistic models and prevalence/risk ratios from log-binomial models would approximate one another. In this study, however, the prevalence of adolescent COVID-19 vaccine hesitancy is above 10% in all sites (and as high as 88% in Dodoma). As a result, the odds ratios would render extreme values and greatly exaggerate the magnitude of associations. Therefore, we believe prevalence ratios from log-binomial models would be more appropriate for this analysis. The following references provide general introductions to the log-binomial models and explain why log-binomial models are preferable to logistic models for non-rare outcomes and prospective study designs. We have now added these references in line 236 (in the track-change version).

David B Richardson, Alan C Kinlaw, Richard F MacLehose, Stephen R Cole, Standardized binomial models for risk or prevalence ratios and differences, *International Journal of Epidemiology*, Volume 44, Issue 5, October 2015, Pages 1660–1672, <https://doi.org/10.1093/ije/dyv137>

Donna Spiegelman, Ellen Hertzmark, Easy SAS Calculations for Risk or Prevalence Ratios and Differences, *American Journal of Epidemiology*, Volume 162, Issue 3, 1 August 2005, Pages 199–200, <https://doi.org/10.1093/aje/kwi188>

ii. Introduction. The introduction has appropriately considered, for clarity of sequence in its content, contextual background, and scoping background (empirical review) but weakly glossing over the theoretical and conceptual clarification in lines 115-116 which is necessary for providing evidence for the needed understanding sought by the study to elucidate the dynamics of the problem phenomenon requiring the study. But the theoretical and conceptual resources required to achieve this has been underplayed in characterizing the science underpinning the problem phenomenon. Theoretical and conceptualization Measurement challenges. Observed lack of sound conceptualization of the problem phenomenon grounded in appropriate behaviour theories since hesitancy just as intention proxy behaviour outcomes has weaken the argument of determinant of Vaccine hesitancy among adolescents.

Where is the review of the theoretical foundation that should provide the explanations of the dynamics of the phenomenon? For this type of study that is contexed in risk of an adverse outcome should be based on the principles of health-seeking explainable from the framework of health belief and concept of prevention. The absence of this is a serious omission to establish the thesis of the study, which renders it superficial and weak.

We thank the reviewer for raising the crucial point regarding the theoretical underpinning for this work. We fully agree that discussion and presentation of the theoretical underpinning will further strengthen this work. To overcome this shortcoming, we have added the discussion on the theoretical underpinning (lines 106-111 in tracked changes): “Based on the health belief model, the likelihood of receiving COVID-19 vaccines may be affected by various factors, such as perceived threats from COVID-19 infection, evaluation of COVID-19 vaccines, cues to action received from the media and other individuals, and modifying sociodemographic characteristics. Behaviors toward vaccines may also be driven by cultural, social, historical, and political factors, which likely vary across cultural and geographical settings.”

We have also created a figure for the conceptual framework based on the health belief model (**Fig 1**). Based on Fig 1 and as added in lines 209-215 in tracked changes: “Data collection and statistical analysis were informed by the theoretical underpinning of the health belief model (**Fig 1**). Based on this conceptual framework, COVID-19 vaccine hesitancy among adolescents may be driven by perceived susceptibility to COVID-19 exposure, perceived severity of COVID-19 infection, perceived effectiveness and safety of COVID-19 vaccines, and cues to action received from media and other influencers (e.g., family members, teachers, healthcare providers). Several individual-level sociodemographic characteristics may also drive willingness to receive COVID-19 vaccines.”

In the section for data collection Line 178 – 186, there seems to be inconsistencies regarding variables in this study. There is mentioned knowledge in Line 179 as a variable measured along with perception and attitudinal disposition, but line 103 omits it and the result does not show a measure for knowledge. The apparent disconnect observed demonstrates the lack of adequate conceptualization and application of an appropriate conceptual framework. These are not appropriately measured in this study and has weakened the presentation of the result and conclusions.

We thank the reviewer for pointing out this apparent inconsistency. We would like to clarify the reason for this apparent disconnect.

We included questions on adolescents' knowledge of the COVID-19 disease (not COVID-19 vaccines, since no vaccines were available at the time) only during Round 1 of the survey but not in the currently reported Round 2. These questions in Round 1 included adolescents knowledge of possible symptoms of COVID-19 infection, transmission methods of COVID-19, and prevention methods against COVID-19. A knowledge score was created summing up adolescents' knowledge of these three domains related to COVID-19. We previously used this score as an outcome in the following article.

Wang D, Chukwu A, Millogo O, Assefa N, James C, Young T, Lankoande B, Workneh F, Hemler EC, Korte ML, Mattei J. The COVID-19 pandemic and adolescents’ experience in sub-Saharan Africa: a cross-country study using a telephone survey. The American journal of tropical medicine and hygiene. 2021 Aug;105(2):331.

For the Round 2 survey, among adolescents who participated in both Rounds 1 and 2, this knowledge score calculated during Round 1 was used as a potential predictor for COVID-19 vaccine hesitancy (Table 3).

During Round 2 of the survey, knowledge of the COVID-19 disease itself was not included in the survey to make room for questions on adolescents’ perceptions and attitudes toward the COVID-19 vaccine. During Round 2, the question on COVID-19 vaccine knowledge was "Have you heard of a vaccine for COVID-19?" The other questions were regarding perceptions and attitudes and have all been presented in the manuscript. The complete questionnaire for Round 2 is publicly available at <https://africa.harvard.edu/files/african-studies/files/arise_covid-19_survey_round_2_adolescent_household_survey_questionnaire.pdf>. We welcome any suggestions on which additional measures on COVID-19 vaccine knowledge, perception, and attitudes can be incorporated in our analyses in ways other than how we present them in the current manuscript.

The statistical analysis is completely flawed by not developing weighted aggregate scores for these variables representing the personal-level chrematistics of the respondents in the various countries and standardizing the resultant means for comparison. This would have been simpler and easily comprehensible, than the complex tables of frequency distribution so difficult to answer the research questions.

We are grateful to the reviewer for this comment. A weighted aggregate score as described by the reviewer would be informative in many settings. However, we believe that the weighted aggregate score may not be the ideal way for analysis and presentation for our study. For the descriptive analyses, we contend that presenting the absolute values of the original variables would be more informative than the standardized values relative to the population mean in each area. The original values of the variables are more intuitive to grasp quickly without having to first understand the distributions in each specific population. Presenting the original values separately by rural and urban areas in each country also enables the comparison between rural and urban settings within each country. For the regression analyses, similarly, we maintain that the original values of the variables seem more appropriate for the analyses as the analyses are pooled across countries, and using values standardized to each country might introduce bias caused by the different underlying distributions for each country/area. Therefore, we thank the reviewer again for this comment and would like to invite the reviewer to reconsider this suggestion regarding weighted aggregate scores.

The results as presented appear very busy. How would the study reconcile the contents of table 1, 2 and 3? It would have been simpler to identify if the sociodemographic characteristics of participants from the rural settings are matched and same for urban setting across countries. The explanatory variables would have been computed as aggregate scores for each country for both rural and urban settings and standardize the mean scores to determine where they are located on the distribution of means of standardized scores.

We thank the reviewer for raising this point. Table 1 summarizes the sociodemographic characteristics of the adolescents included in the survey, Table 2 presents the adolescents’ willingness to receive COVID-19 vaccines (i.e., attitudes), and Table 3 (currently S2 Table) describes the adolescents' perceptions of the safety, effectiveness, and side effects of the vaccines (i.e., perceptions). Therefore, we believe that the content presented in Tables 1-3 does not overlap with or contradict one another.

We agree with the reviewer that we have a relatively large number of tables, making the presentation appear busy. Therefore, we have decided to move the previous Table 3 (perceptions of COVID-19 vaccine) to the supplementary material as S2 Table and the previous Table 4 (self-perceived determinants of COVID-19 vaccine willingness) to the supplementary material as S3 Table. The numbering of the other tables in the main file and the supplementary materials has been updated accordingly.

We thank the reviewer again regarding the point of using weighted aggregate scores. Please kindly refer to our response to the bullet point above regarding the use of weighted aggregate scores.

Discussion: It would have been very appropriate to reiterate the purpose of the study as an introduction for the discussion before presenting findings. It would be better to use the term "associated with" than use "influenced" in line 348, 404 and elsewhere because this cannot be determined by the study design implemented. Revise where the word "influence" has been used accordingly except in the context of likelihood.

We thank the reviewer for the good suggestion to reiterate the aim of the study before presenting findings. We have revised the first paragraph of the Discussion section accordingly (lines 387-389 in tracked changes). We have also made the suggested revision on wording everywhere when “influenced” was used.

Line 352: Note the use of the word "level" implies measurement requiring scale of measures involving scoring the variables constituting the determinants particularly if they are in the domain of interval scale or ratio scales.

We thank the reviewer for pointing this out. We had used the word "level" loosely to indicate "prevalence." We should have been more careful with the wording and have now revised “level” to “prevalence” throughout the manuscript (including in the title) to be more precise in terminology.

Reviewer #2: PLOS GLOBAL PUBLIC HEALTH

LEVELS AND DETERMINANTS OF COVID-19 VACCINE HESITANCY AMONG SUB-SAHARAN AFRICAN ADOLESCENTS.

This article by Dongqing et al, focused on covid-19 vaccine hesitancy among adolescents in a cross-country survey to determine reasons for hesitancy and offer recommendations or solutions to the challenges encountered in the global efforts to control the covid-19 pandemic. This paper reported that the adolescents constitute about 23% of the population of sub-Saharan Africa. (line 88), with a mortality and morbidity from corona virus found to be lower than in adults. This paper opined that there was a strong rational for providing covid-19 vaccines to adolescents because infected adolescents could transmit the virus to other individuals as well as develop severe symptoms or complications hence the need for vaccination.

This article is very appropriate and timely as it addresses a fundamental global problem that has affected the human race with a view to providing a solution to the challenge posed by the corona virus pandemic in sub-Saharan Africa.

We thank reviewer 2 for the time and effort in reviewing our study and the kind words on the manuscript. The reviewer's comments and suggestions are valuable, and we hope we have adequately addressed the reviewer's inputs. Please find below our point-by-point responses to the reviewer’s suggestions.

THE TITLE: The title is very appealing and appropriate. It would have been more explicit if the authors distinguished the words "levels "and "Determinants" in more concise terms with detailed analysis inside the article.

We thank the reviewer for this important suggestion on the title. We have revised the title as “COVID-19 vaccine hesitancy and its determinants among sub-Saharan African adolescents.” In this way, the element of “levels” is implied, and the element of "determinants" is better highlighted. We welcome any feedback on the appropriateness of the new title and would be delighted to revise further.

THE ABSTRACT: The abstract was able to summarize concisely the research that was done with an in-depth display of the essential information required.

We thank the reviewer for this helpful evaluation of the Abstract.

INTRODUCTION: The authors were able to give an over view of the pandemic, the different vaccines and why the adolescents needs vaccination.

We appreciate the reviewer’s helpful evaluation of the Introduction section.

METHODOLOGY: This article presented a unique study design, study population, data collection and statistical analysis. But there are some points that will require further clarifications:

We thank the reviewer for the kind words on our methodology and are grateful for the reviewer’s valuable suggestions for further clarification and revision. Please kindly find attached the point-by-point responses to the feedback.

1. The authors reported this article as “an ongoing survey that used a novel mobile platform and computer-assisted telephone interviewing to collect data from Sub-Saharan African adolescents, adults and healthcare providers’’. ( line122). Is it still ongoing or concluded?

We thank the reviewer for this point. The study is indeed concluded now, and no further rounds of data collection are planned. We used the word "ongoing" to indicate that the study can be considered a continuation of our Round 1 effort. To avoid confusion, we have removed the word "ongoing" from line 129 in tracked changes.

2. The authors need to throw more light on the computer-assisted telephone interviewing data collection instrument bearing in mind that the sub-Saharan African countries involved in the study are developing countries with adolescents having little or no access to computers or phones in rural and urban areas.

We thank the reviewer for this very helpful suggestion. We agree that how we reached the adolescents in the sub-Saharan African settings warrants further clarification. Data collectors called each selected household to obtain consent to survey the household head or another adult member in the household. The data collectors then obtained parental or guardian consent to speak to the adolescent through the adult household member’s phone if an adolescent was present in the household. If the adolescent was not present in the household (e.g., the adolescent was in school), an arrangement was made to call back at a later time. When contact was made with the adolescents through the parent’s phone, the data collectors obtained assent directly from the adolescents and completed the adolescent interview. We have added this description in tracked changes in lines 153-159.

3. In addition, adolescent range from 10-19 years. Considering the social economic factors listed in this article, these adolescents may not be able to decipher the different types of vaccines. Parents may also likely be the major determinants of their acceptance or rejection of the vaccines. Kindly clarify how these adolescents were able to decide on their own to accept or reject the vaccine. Adolescents within the ages of 18-19 can be legally considered as adults who can take decisions for themselves. Adolescents between 10-17 years which represent a large percentage of your study are legally not able to decide for themselves to accept or reject covid-19 vaccines. I believe that a strict categorization of these adolescents into smaller age brackets would have provided a more reliable data for this study. Willis et al and Afifi et al as referenced in your article demonstrated these sub-divisions. ( lines 530,535).

We thank the reviewer for this important suggestion. We agree that a more granular assessment of COVID-19 vaccine hesitancy by age would be informative. We have examined the levels of COVID-19 vaccine hesitancy by every single age (e.g., 10, 11, 12…19 years) and also by the age bracket of young adolescents (10-14 years) versus older adolescents (15-19 years). We presented these additional analyses in **S1 Table** and described the results in lines 269-270 in tracked changes. This stratified analysis by every single age and age bracket did not find consistent trends in COVID-19 vaccine hesitancy by age, which is consistent with the weak association between age and vaccine hesitancy in Table 3. This finding is also consistent with Willis et al. and Afifi et al., neither of which reported a significant difference in COVID-19 vaccine hesitancy by age in high-income settings.

4. Furthermore, a sample size of 300 households showed the data was collected from adolescents living in these households. Considering that this study was conducted between July and December, 2021 which falls into the school calendar. Adolescents are expected to be in school because the adolescent age is a school age for children and young adults. Kindly clarify how you reached these adolescents in both urban and rural areas. How was data collected from uneducated adolescents in rural/urban areas?

We thank the reviewer for the great questions. The reviewer rightly pointed out that the window of data collection overlapped with the academic year in some countries. As we clarified in our response to comment #2, if the adolescent was not present in the household (e.g., the adolescent was in school), an arrangement was made to call back at a later time. When contact was made with the adolescents through the parent’s phone, the data collectors obtained assent directly from the adolescents and completed the adolescent interview. We have now clarified this in tracked changes in lines 153-159. As data collection with the adolescents was conducted through parents’ or guardians’ phones, we were able to cover out-of-school adolescents in both rural and urban areas. This did result in the limitation that, due to the nature of phone-based interviews, the adolescents included in most areas were those who resided in households with access to a mobile phone. Consequently, the sample might underrepresent adolescents from under-resourced households, which may affect generalizability, which we acknowledged in lines 498-501 in the track-change version.

5. There are several countries in sub-Saharan Africa, the authors may wish to clarify how they determined the samples and sizes more scientifically.

We thank reviewer 2 for raising this important point. The study countries and study sites were selected based on research team capacity and infrastructure from existing collaborations in the African Research, Implementation Science and Education (ARISE) Network. The target sample size was determined based on budget constraints, and we did not conduct a formal sample size calculation to determine the target sample size. We have now clarified this in tracked changes in lines 147-151.

6. In the study, data was collected from those above the defined adolescent age. The study reported in some cases data was collected from adolescents aged 20years as at the time of round 2 data collection (line 160,161). I believe the authors could have maintained the 10-19 years adolescent age range.

We thank the reviewer for this comment. We also agree that the age range can be maintained with 10-19 years with minimal loss of participants and better clarity. We have therefore removed adolescents aged 20 in the current survey round from all analyses. Due to this small yet major change to the study sample, we have updated all the results with the new sample size. As a result, almost all of the specific numbers in the tables and many of the numbers in the text have been updated.

7. Repeated references was alluded to "the previous round 1 survey" and “ the details of the study design and study population in round 1 described previously(lines 151, 178,205,206)”. Is this study a continuation of another research?

We thank the reviewer for this important question. In certain ways, this survey may be considered a continuation of a previous survey (Hemler et al. The American journal of tropical medicine and hygiene. 2021 Aug;105(2):310.). The Round 1 and Round 2 surveys share the following similarities.

a. Overlap in study sites: both rounds had the same rural and urban sites in Burkina Faso, Ethiopia, and Nigeria and used the same infrastructure of computer-assisted telephone interviewing.
b. Both rounds had a similar focus on the impacts of the COVID-19 pandemic on sub-Saharan Africa.
c. Both rounds had three groups of participants: adolescents, adults, and healthcare providers.
d. We attempted in Round 2 to get in touch again with the Round 1 participants to respond to the Round 2 survey questionnaires.

Round 2 also differed from Round 1 in the following ways.

a. New study countries, Tanzania and Ghana, were added in Round 2.
b. The surveys used in Rounds 1 and 2 are not identical and differ in important ways. The focus of Round 2 was on COVID-19 vaccine perceptions and attitudes and COVID-19 vaccine hesitancy. In contrast, Round 1 focused on the knowledge, perceptions, and attitudes of the COVID-19 pandemic and a more detailed evaluation of its impacts on daily lives.
c. The proportion of adolescents who participated in both rounds was modest (In Burkina Faso, Ethiopia, and Nigeria, adolescents who also participated in the Round 1 survey varied from 6% in Lagos to 63% in Addis Ababa).

Therefore, although Round 2 can be considered a continuation of Round 1 research, it also represents a standalone research project. To provide an independent outlook for the study, we have reduced references to Round 1 as much as possible, and the references and descriptions of Round 1 now appear in the Methods section but not elsewhere.

8. To give this study an independent outlook, I believe the authors could have concisely outlined the relevant information from “the round 1” which was relevant to round 2 to avoid confusion due to incessant referrals to read up a previous article.

We thank reviewer 2 for raising this crucial point. We agree that the paper should be able to stand by itself without having to refer the readers to another article. As noted in our response to the above comment, Rounds 1 and 2 differ in important ways, so repeated reference to the Round 1 paper is unnecessary. Therefore, to give an independent outlook for the study, we have reduced references to Round 1 as much as possible, and the reference and descriptions of Round 1 now appear in the Methods section and not elsewhere.

9. A clearly outlined aims and objectives at the beginning of the study could further complement the discussions and conclusions from this study.

We thank the reviewer for this great suggestion. We have added the specific aims in tracked changes in lines 387-389.

10. The authors identified three major limitations of this study (lines 442-453). These give the impression that the study areas, sample size determination and data collected did not give a robust representation of the true picture of the covid-19 hesitancy among adolescents in sub-Saharan Africa.

We thank reviewer 2 for this important comment. We agree with the reviewer that this is a key limitation of the study, as we acknowledged in lines 491-494. Because the study areas were selected based on existing connections and infrastructure, and the adolescents were not selected probabilistically, the results from this study may not be generalized to all adolescents in the country. Nevertheless, we attempted to increase the generalizability by calling adolescents randomly from each sampling frame (lines 146-147). The generalizability of the sample was also increased by including countries and areas geographically spread across sub-Saharan Africa (lines 494-496). Therefore, we hope the reviewer will agree that our analysis nonetheless represents an important (albeit imperfect) characterization of COVID-19 vaccine hesitancy among sub-Saharan African adolescents (lines 496-497).

CONCLUSION: I agree with the authors that the strength of this article is the inclusion of different cities from multiple countries in sub-Saharan Africa and the attempt to show a wide array of the potential determinants of Covid-19 vaccine hesitancy among adolescents. In addition, the claim by the authors that this study is the first of its kind to examine the levels and determinants of covid-19 vaccine hesitancy among adolescents in multiple countries from sub-Saharan Africa makes this study unique and novel.

Finally, this article shows a massive work done with good editing and outlay of data with statistical analysis. The study data as presented by the authors clearly support the conclusions drawn by the study.

We thank reviewer 2 for the kind words on our manuscript. We are excited that the reviewer finds our study unique, novel, and contains a massive amount of work. We are again grateful to reviewer 2 for the time and effort in providing valuable suggestions on our manuscript. We hope the reviewer will find that we have adequately incorporated the suggestions and welcome any additional input.
